# Supplementary material for: Bacterial diversity and antibiotic susceptibility profile of the isolates from public vehicles in Butwal Sub-Metropolitan City, Rupandehi, Nepal
Source: PLOS Glob Public Health. 2026 May 8;6(5):e0006421. doi: 10.1371/journal.pgph.0006421 (PMC13155621; doi:10.1371/journal.pgph.0006421)
Supplement: Attachment — Submitted filename: Comments- Manuscript PGPH-D-25-01435.docx [file pgph.0006421.s002.docx]

**Comments for the authors**

Dear Authors, please find below my comments and suggestions aimed to improving your manuscript

The study addresses a relevant and current topic with significant impact on public health. However, some aspects require greater clarity.

The manuscript does not include continuous line numbering throughout the content. I recommended that the authors add line numbers in accordance with the journal’s guidelines and to facilitate the identification of specific sections.

The statement “data available upon request from the corresponding author” does not meet PLOS data policy requirements. I request that the authors deposit the data in a trusted, policy accessible repository and update the Data Available section accordingly.

There are spelling and grammatical mistakes, a careful review is advised.

**Abstract**

Line 9: explain briefly how public transportation systems can serve as breeding grounds for bacterial contamination.

Line 24 “in the conclusion” mention the concerning scenario of antimicrobial resistance to antibiotics found in the study, as this increases the risks to public health.

**Introduction**

**Line 34 “***studies conducted across different regions****…”*** I recommend that, for enhanced clarity and contextualization, the specific location where the referenced study was conducted be clearly indicated.

**Line 52:** the passage “A *previous study has found…”*  references [11-14] were cited. Could the authors clarify how multiple references were associated with single study? I suggest that the authors revise this section and cite only the reference that specifically corresponds to the study in question.

Please also indicate the location where the study was conducted.

**Materials and Methods**

**Line 73** we kindly request clarification regarding the period from June to February 2022. From June (year) to February 2022.

**Line 100:** the citation is not consistent with the reference to the manual in question. I recommend revising the citation, as it appears to correspond to reference [19].

**Results**

**Line 136:** over one year? How? From June to February? Please clarify the statement.

**Line 153:** do not included figures within the main text. figures/graphs should be submitted s separate files. please refer to the specific guidelines provided by PLOS.

**Line 160**: the value of (N) does not correspond the percentages presented. Shouldn’t it be AC Buses 14 (19.44%) and Winger 26 (36.11%)?

The same applies to **line 162**. AC Buses n=20 relates to 38.46% and 10 relates to 19.23%.

**Lines 163 a****nd 164:** in my opinion, it is not essential to report values that were not statistically significant- only those that reached statistical significance.

**in the table**: was *Citrobacter species* not significant? Based on the results presented in the table, it appears to be statistically significant.

**Line 183:** I suggest that the information be presented in table format to enhance clarity.

**Table 2:** it is advisable to specify the meanings of the abbreviations (S/R) at the bottom of the table.

**Discussion**

The discussion section still requires further improvement, especially regarding the accuracy and consistency of the citations. I recommend a more rigorous review.

The first paragraph of the discussion should include a concise summary of the main findings, clearly indicating whether the study objectives were achieved, highlight their initial implications for public health.

Each time a study is mentioned, it is advisable to specify the location where it was conducted and highlight its main findings.

For the citation of **Kwan *et al.,*** please specify the year in which the study was conducted. Additionally, it would be important to include the main findings (particularly the percentage of Gram-positive bacteria identified in their study). It is also recommended that the authors provide a possible explanation for the prevalence of Gram-positive bacteria found in their own study, in comparison to Kwan *et al.,*

Kindly note that this citation (Kwan *et al.*,) does not appear in the reference list. I recommend reviewing the references to ensure all cited works are properly listed.

It is noted that most of the in-text citations do not correspond to the references listed. For example, the study conducted by **Gimmanco *et al.,*2012** is cited as reference [23] in the discussion, but in the reference list, this study actually corresponds to citation [18] not [23].

Please specify the year for all citations mentioned in the discussion section, **Lam et al., (year), Eibach et al., (year).**

I suggest following the same structure when presenting your findings: start with your main result, then discuss similar /different results found in the other studies, provide a possible explanation or reason for your findings, and finally, highlight the implications for public health.

**Passage** “Recent research supported these findings…” which studies are these? Please clarify.

**Passage** “These findings align with those from other studies [27-30]”. What do these studies say?

**Passage** “These findings were supported by recent research”. Discuss the findings of these recent studies.

Present the study limitations at the end of the discussion section, and discuss them.

What policy strategies can be adopted to improve the situation? Please present them in the form of recommendations.

**Conclusion**

The conclusion needs to be improved and more elaborated.

**References**

Same references cited in the manuscript cannot be found in recognized scientific databases. For instance, the case of Beavogui *et al*., (2024). I recommend verifying the existence and accessibility of these sources. Please be reminded that the reference list should include only published or accepted works. Abstracts from conferences, unpublished manuscripts, or personal communications should not be included.

The DOI’s presented for references 2,3, 22, 24 and 26 lead to articles different from those described in the references list. I recommend that the authors verify the accuracy of the references and update the information according (DOI, title, authors and journal).

**Reference 26**: the manuscript cites DOI ’10.1093/cid/ciu465’ for the reference titled “Antimicrobial susceptibility testing: A review of general principles and contemporary practices”. However, this DOI does not correspond to any indexed article and appears to be incorrect. The correct DOI for this reference is <https://doi.org/10.1086/513938> published in 1998. It is advisable to verify this reference carefully for accuracy.

For references **4**, **7 and 12**, I suggested verifying and correcting the author’s names according to the original publication, in order to ensure accuracy and proper attribution of authorship.
