# Supplementary material for: Bacterial diversity and antibiotic susceptibility profile of the isolates from public vehicles in Butwal Sub-Metropolitan City, Rupandehi, Nepal
Source: PLOS Glob Public Health. 2026 May 8;6(5):e0006421. doi: 10.1371/journal.pgph.0006421 (PMC13155621; doi:10.1371/journal.pgph.0006421)
Supplement: Attachment — Submitted filename: Response to Reviewers.docx [file pgph.0006421.s004.docx]

**8/27/2025**

**To:** The Editor
**PLOS Global Public Health**

**Subject:** Rebuttal Letter – Manuscript No: PGPH-D-25-01435

Dear Editor,

We sincerely thank you and the reviewers for the constructive and insightful comments on our manuscript entitled “Bacterial Diversity and Antibiotic Susceptibility Profile of the Isolates from Public Vehicles in Butwal Sub-Metropolitan City, Rupandehi, Nepal.” The feedback has been invaluable in improving the clarity, rigor, and overall quality of our work.

We have carefully revised the manuscript in line with all suggestions. Below, we provide a point-by-point summary of the major revisions.

### ****Reviewer #1: Revisions****

**Comment 5 (Line 139-Sampling strategy):**

The authors state that swab samples were collected from different parts of the buses. It would strengthen the manuscript to explain the rationale for selecting these particular sites.
**Response:** We agree and have added an explanation. The selected sampling sites (steering wheel, passenger handles, seat handles, and seat backs) were chosen because they represent **high-touch surfaces frequently contacted by drivers and passengers**, making them most likely to harbor bacterial contamination.

**Comment 6 (Line 142 – Graph of gram-negative bacteria):**
It is suggested that the bacterial frequency values be reordered from lowest to highest (or vice versa), for consistency and improved readability, as was done with gram-positive bacteria.
**Response:** We have reordered the bacterial frequency values for gram-negative isolates from lowest to highest for **better readability and consistency**.

**Comment 7 (Graph design):**
In the graph showing the distribution of bacteria, it would be clearer to use distinct colors to differentiate between sampling locations such as the steering wheel and seat handle.
**Response:** We have revised the graphs in table format as a feedback provided by reviewers 2.

**Comment 8 (Results section – Images):**
While the manuscript describes the bacterial analysis, no images or visual documentation of the findings are provided. Including representative images (e.g., culture plates or microscopic images) would add value and help validate the results.
**Response:** Representative images of **culture plates and Gram-stained microscopic views** have been added as supplementary figures to visually support the reported findings.

**Comment 9 (Ethics approval):**
Although the authors state that ethical approval was obtained from the Pokhara University Research Center, the ethics approval number is missing.
**Response:** We have now included the **ethical approval number: PURC Grant No. 02-2079/80** in the Ethics Statement section.

**Comment 10 (References):**
Several references require formatting corrections for Vancouver style.
**Response:** We have carefully revised and reformatted all references according to the **Vancouver style** required by the journal, ensuring accuracy in journal titles, article titles, punctuation, and DOI/URL presentation.

### ****Reviewer #2: Revisions****

- **Line numbering:** Continuous line numbering has been added as per journal guidelines.
- **Data availability:** All datasets have been deposited in [repository name, e.g., Zenodo/Harvard Dataverse] with an accessible link, and the Data Availability Statement has been updated.
- **Language editing:** The manuscript was thoroughly revised to correct spelling, grammar, and style issues.
- **Keywords:** Updated to standardized **MeSH terms**.

**Abstract:**

- Line 9: Expanded to briefly explain how public transport systems serve as potential reservoirs for bacterial contamination.
- Line 24: Conclusion revised to emphasize antimicrobial resistance as a major public health concern.

**Introduction:**

- Line 34: Clarified the specific location of the referenced study.
- Line 52: Removed multiple references linked to a single study and corrected citation to match study location.

**Materials and Methods:**

- Line 73: Clarified the study period as June 2021 to February 2022.
- Line 100: Corrected the reference to ensure consistency with the manual cited.

**Results:**

- Line 136: Clarified statement regarding study duration.
- Line 153: Figures moved to supplementary files in compliance with journal policy.
- Lines 160–162: Corrected inconsistencies between “N” values and percentages.
- Lines 163–164: Removed non-significant results, retaining only statistically significant findings.
- Citrobacter spp.: Corrected to reflect statistical significance.
- Line 183: Data reformatted into a table for clarity.
- Table 2: Abbreviations (S = Sensitive, R = Resistant) defined in footnotes.

**Discussion:**

- Restructured for logical flow: findings are now followed by comparative evidence, explanations, and implications.
- Opening paragraph revised to summarize key findings and link to objectives.
- Outdated or inaccessible references (e.g., Kwan et al., Beavogui et al.) removed and replaced with recent indexed studies.
- In-text citations corrected to match updated reference list (e.g., Gimmanco et al., 2012).
- Vague statements revised with specific updated studies.
- A section on **study limitations** was added.
- Policy recommendations for hygiene and antimicrobial resistance reduction in public transport were incorporated.

**Conclusion:**

- Expanded to synthesize findings, emphasize public health implications of contamination and antimicrobial resistance, and highlight the need for policy-level interventions.

**References:**

- All references verified with PubMed, CrossRef, and other scientific databases.
- DOIs corrected and validated (All references are reformatted)
- Author names corrected in references 4, 7, and 12.
- In-text citations and reference list made consistent.

In conclusion, we have thoroughly revised the manuscript in response to all reviewer and editor comments. The updated version now includes corrected references, standardized MeSH keywords, improved discussion, expanded conclusion, inclusion of supplementary images, and overall strengthening of weak sections. We believe these revisions have substantially enhanced the quality and impact of the manuscript.

We once again thank the reviewers and the editorial team for their valuable time and constructive feedback, and we respectfully request reconsideration of our manuscript for publication in PLOS Global Public Health.

Sincerely,
**Ram Bahadur Khadka** (Corresponding Author)
On behalf of all co-authors
