# Supplementary material for: Bacterial diversity and antibiotic susceptibility profile of the isolates from public vehicles in Butwal Sub-Metropolitan City, Rupandehi, Nepal
Source: PLOS Glob Public Health. 2026 May 8;6(5):e0006421. doi: 10.1371/journal.pgph.0006421 (PMC13155621; doi:10.1371/journal.pgph.0006421)
Supplement: Attachment — Submitted filename: Rebuttal Letter Response to Reviewer 1.docx [file pgph.0006421.s005.docx]

**10/31/2025**

**To:** The Editor
**PLOS Global Public Health**

**Subject:** Rebuttal Letter – Manuscript No: PGPH-D-25-01435

Dear Editor and Reviewer # **1***,*

We sincerely thank you and the reviewers for the constructive and insightful comments on our manuscript entitled **“Bacterial Diversity and Antibiotic Susceptibility Profile of the Isolates from Public Vehicles in Butwal Sub-Metropolitan City, Rupandehi, Nepal.”** The feedback has been invaluable in improving the clarity, rigor, and overall quality of our work. Additionally, the **PLOS NAAS tool** was applied to assess **Figure 1**, and the figure has been **uploaded in accordance with NAAS requirements.**

We have carefully revised the manuscript in line with all suggestions. Below, we provide a point-by-point summary of the major revisions.

### ****Reviewer #1: Revisions****

**Comment 1 (Point 139 – Sampling Site Justification):**
The reviewer noted that the manuscript did not justify the choice of sampling locations.

**Response:**
We have added the following sentence for clarification:

“These locations were chosen because they are the surfaces most frequently touched by passengers and drivers, thereby presenting the highest risk of bacterial contamination due to frequent contact.”
This sentence has been incorporated at Lines 140–142.

**Comment 2 (Point 142 – Graph Arrangement):**
The reviewer recommended arranging the Gram-negative bacteria frequency data in ascending order for better organization.

**Response:**
The frequency data for Gram-negative bacteria have been reorganized from lowest to highest values to ensure consistent presentation and improved readability.

**Comment 3 (Graph Design):**
The reviewer suggested using distinct colors to differentiate between sampling locations such as the steering wheel and seat handle.

**Response:**
We have revised the graphical representation into a **table format** following feedback from Reviewer #2. This change improves clarity and readability; therefore, color-coding was not applied.

**Comment 4 (Results Section – Supporting Images):**
The reviewer requested inclusion of images to support the bacterial analysis results.

**Response:**
Representative images of bacterial isolates have been added as **supplementary materials (Annexes)**, uploaded separately according to journal guidelines.

**Comment 5 (References):**
The reviewer identified minor duplications and inconsistencies in reference links.

**Response:**
All reference duplications and link inconsistencies have been corrected.

We once again thank the reviewers and the editorial team for their valuable time and constructive feedback, and we respectfully request reconsideration of our manuscript for publication in PLOS Global Public Health.

Sincerely,
**Ram Bahadur Khadka** (Corresponding Author)
On behalf of all co-authors
