# Supplementary material for: Bacterial diversity and antibiotic susceptibility profile of the isolates from public vehicles in Butwal Sub-Metropolitan City, Rupandehi, Nepal
Source: PLOS Glob Public Health. 2026 May 8;6(5):e0006421. doi: 10.1371/journal.pgph.0006421 (PMC13155621; doi:10.1371/journal.pgph.0006421)
Supplement: Attachment — Submitted filename: Rebuttal Letter Response to Reviewer 2.docx [file pgph.0006421.s006.docx]

**11/30/2025**

**To:** The Editor
**PLOS Global Public Health**

**Subject:** Rebuttal Letter – Manuscript No: PGPH-D-25-01435

Dear Editor and Reviewer **#2,**

We sincerely thank you and the reviewers for the constructive and insightful comments on our manuscript entitled **“Bacterial Diversity and Antibiotic Susceptibility Profile of the Isolates from Public Vehicles in Butwal Sub-Metropolitan City, Rupandehi, Nepal.”** The feedback has been invaluable in improving the clarity, rigor, and overall quality of our work.

We sincerely thank the reviewer for taking the time to re-evaluate our revised manuscript. We deeply appreciate the constructive feedback provided in the previous round, which has significantly helped us improve the quality and clarity of our work.

We have carefully revised the manuscript in line with all suggestions. Below, we provide a point-by-point summary of the major revisions.

### ****Reviewer #2: Revisions****

**1. Comment: Missing continuous line numbering**

**Reviewer:**
“In the previous version, the manuscript had continuous line numbering, but it was not applied throughout the entire document. In the current version, there is no line numbering at all, which makes the revision process difficult. I kindly request that the authors apply continuous line numbering to the entire manuscript.”

**Response:**
We thank the reviewer for pointing this out.
Continuous line numbering has now been applied throughout the entire manuscript, including the title page, abstract, main text, references, and figure legends.

**2. Comment: Specify exactly in which repository the database is available, and provide access link**

**Reviewer:**
“Please specify exactly in which repository the database is available, and, if possible, provide the access link to the database.”

**Response:**
We have updated the Data Availability Statement accordingly.
**All underlying data have been deposited in the Zenodo public repository**, and the dataset is freely accessible at the following DOI link: <https://doi.org/10.5281/zenodo.17766523>

This is now clearly stated in the revised manuscript (Data Availability section).

**3. Comment: Discrepancy in study period (June 2023–July 2024 vs. June 2021–February 2022)**

**Reviewer:**
“Review the materials and methods section and clarify whether the study period was from June 2023 to July 2024, or from June 2021 to February 2022 as stated in the response letter. There is discrepancy between the two descriptions.”

**Response:**
We appreciate this important observation.
The correct study period is: June 2023 to July 2024.

We have now corrected and harmonized this timeline **throughout the entire manuscript**, including the Materials and Methods section and the response letter. All inconsistent statements have been removed.

**4. Comment: Reference list requires attention and clarity**

**Reviewer:**
“Review the reference list, as it requires more attention and clarity.”

**Response:**
Thank you for highlighting this issue.
We have now thoroughly revised the entire reference list.

We once again thank the reviewers and the editorial team for their valuable time and constructive feedback, and we respectfully request reconsideration of our manuscript for publication in PLOS Global Public Health.

Sincerely,
**Ram Bahadur Khadka** (Corresponding Author)
On behalf of all co-authors
